# Proline-rich antimicrobial peptide, PR-39 gene transduction altered invasive activity and actin structure in human hepatocellular carcinoma cells

**DOI:** 10.1038/sj.bjc.6690707

**Published:** 1999-10

**Authors:** T Ohtake, Y Fujimoto, K Ikuta, H Saito, M Ohhira, M Ono, Y Kohgo

**Affiliations:** Third Department of Internal Medicine, Asahikawa Medical College, Nishikagura 4–5, Asahikawa, 078-8307, Japan

**Keywords:** antimicrobial peptide, PR-39, actin, hepatocellular carcinoma, syndecan

## Abstract

PR-39 is an endogenous proline-rich antimicrobial peptide which induces the synthesis of syndecan-1, a transmembrane heparan sulphate proteoglycan involved in cell-to-matrix interactions and wound healing. Previously, we revealed that the expression of syndecan-1 was reduced in human hepatocellular carcinomas with high metastatic potential and speculated that syndecan-1 played an important role in inhibition of invasion and metastasis. It is assumed that a modification of this process with PR-39 and syndecan-1 may result in a new strategy by which it can inhibit the invasion and metastasis. Therefore, we transduced a gene of PR-39 into human hepatocellular carcinoma cell line HLF, which shows a low expression of syndecan-1 and a high in vitro invasive activity, and examined whether this procedure could reduce the invasive activity of tumour cells. In two transfectants with PR-39 gene, the syndecan-1 expression was induced and the invasive activity in type I collagen-coated chamber was inhibited. Moreover, these transfectants showed the suppression of motile activity assayed by phagokinetic tracks in addition to the disorganization of actin filaments observed by a confocal imaging system. In contrast, five transfectants with syndecan-1 gene in the HLF cells revealed suppression of invasive activity but did not alter the motile activity and actin structures of the cell. These results suggest that PR-39 has functions involved in the suppression of motile activity and alteration of actin structure on human hepatocellular carcinoma cells in addition to the suppression of invasive activity which might result from the induction of syndecan-1 expression. © 1999 Cancer Research Campaign


*British Journal of Cancer* (1999) **81**(3), 393–403
© 1999 Cancer Research Campaign
Article no. bjoc.1999.0707

### Proline-rich antimicrobial peptide, PR-39 gene transduction altered invasive activity and actin structure in human hepatocellular carcinoma cells

**T Ohtake, Y Fujimoto, K Ikuta, H Saito, M Ohhira, M Ono and Y Kohgo**

Third Department of Internal Medicine, Asahikawa Medical College, Nishikagura 4–5, Asahikawa 078-8307, Japan

**Summary** PR-39 is an endogenous proline-rich antimicrobial peptide which induces the synthesis of syndecan-1, a transmembrane heparan sulphate proteoglycan involved in cell-to-matrix interactions and wound healing. Previously, we revealed that the expression of syndecan-1 was reduced in human hepatocellular carcinomas with high metastatic potential and speculated that syndecan-1 played an important role in inhibition of invasion and metastasis. It is assumed that a modification of this process with PR-39 and syndecan-1 may result in a new strategy by which it can inhibit the invasion and metastasis. Therefore, we transduced a gene of PR-39 into human hepatocellular carcinoma cell line HLF, which shows a low expression of syndecan-1 and a high in vitro invasive activity, and examined whether this procedure could reduce the invasive activity of tumour cells. In two transfectants with PR-39 gene, the syndecan-1 expression was induced and the invasive activity in type I collagen-coated chamber was inhibited. Moreover, these transfectants showed the suppression of motile activity assayed by phagokinetic tracks in addition to the disorganization of actin filaments observed by a confocal imaging system. In contrast, five transfectants with syndecan-1 gene in the HLF cells revealed suppression of invasive activity but did not alter the motile activity and actin structures of the cell. These results suggest that PR-39 has functions involved in the suppression of motile activity and alteration of actin structure on human hepatocellular carcinoma cells in addition to the suppression of invasive activity which might result from the induction of syndecan-1 expression. © 1999 Cancer Research Campaign

**Keywords:** antimicrobial peptide; PR-39; actin; hepatocellular carcinoma; syndecan

PR-39 is an endogenous antimicrobial peptide isolated from pig small intestines (Agerberth et al, 1991; Storici and Zanetti, 1993); it has been recognized as an important mediator of innate immunity against microbes (Boman, 1995; Martin et al, 1995). Among antimicrobial peptides, PR-39 has been classified into a proline- and arginine-rich family, which also includes Bac5 and Bac7 of bovine neutrophils (Storici et al, 1994). Most antimicrobial peptides interact with bacterial membranes and cause lysis, but PR-39 kills bacteria by a non-pore-forming mechanism, presumably by inhibiting DNA and protein synthesis (Boman et al, 1993). To date, there is no other antimicrobial peptide of animal origin known to have a similar mechanism of action (Agerberth et al, 1996). The pre-proform of PR-39 consists of three parts: a signal sequence (preregion) for targeting to the endoplasmic reticulum, a proregion which belongs to the cathepsin L inhibitor (cathelin) family, and a mature region composed of 49% proline and 24% arginine. Recently, new functions of PR-39 have been reported that PR-39 inhibits phagocyte NADPH oxidase activity of pig neutrophils by binding to Src homology 3 (SH3) domains of $p47^{phox}$ (Shi et al, 1996), and that PR-39 binds to and affects a SH3-containing signal transduction protein, P130Cas (Chan and Gallo, 1998). Other proline-rich peptides bind various intracellular SH3 domain-containing proteins, so that proline-rich motif of PR-39 may have a number of functions affecting a variety of intracellular signalling pathways and cytoskeletal proteins, in a range of organisms from yeast to man (Yu et al, 1994; Pawson, 1995). In an attempt to isolate the human counterpart of PR-39, Agerberth et al found FALL-39, the proregion of which belonged to the cathelin family, while its mature region did not contain proline-rich sequences (Agerberth et al, 1995). Thus, the proline-rich region of PR-39 is thought to be essential for all its functions. However, in the human counterpart containing proline, arginine sequences have not yet been found.

Independently, while trying to isolate factors for promoting wound healing by examinig the ability to induce syndecan-1 expression in vitro, Gallo et al (1994) found a 4.7 kDa protein from pig wound fluids. They initiated their work because syndecans are cell surface proteoglycans involving cell-to-matrix interactions and growth factors binding in mouse mesenchymal cells and are essential for wound healing (Gallo et al, 1994). By amino acid analysis, it was found that this syndecan-inducing factor was identical to PR-39.

Moreover, we found that the expression of syndecan-1 was reduced in human hepatocellular carcinomas (HCC) with high metastatic potential and speculated that syndecan-1 played an important role in inhibition of invasion and metastasis (Matsumoto et al, 1997). Our speculation was supported by Liebersbach's paper that the transfection of syndecan-1 gene inhibited invasion into type I collagen by the cell binding in human B lymphoid cell line (Liebersbach and Sanderson, 1994). Like most malignancies, the leading cause of death from this carcinoma still results from intrahepatic and/or distant metastasis, even though many advanced therapeutic procedures, both surgical and interventional, have been made. Therefore, it is assumed that a modification of this process might become a new strategy for inhibiting the invasion and metastasis of tumour cells.

*Received 10 November 1998*
*Revised 22 April 1999*
*Accepted 22 April 1999*

*Correspondence to:* Y Fujimoto

In the present study, we investigated whether or not the PR-39 gene transfection into human HCC cells could suppress the invasive activity, because PR-39 may have multifunctions affecting cell adhesion and intracellular signaling directly or indirectly. The possibility of the PR-39 gene as a candidate for a new strategy of gene therapy was evaluated.

### MATERIALS AND METHODS

### Cell culture

Human HCC cell lines, HuH-1 and HuH-2 (Huh and Utakoji, 1981) were given by Eisai Laboratory (Tsukuba, Japan), and HLE and HLF (Doi et al, 1975) were purchased from Japanese Cancer Research Resources Bank (Tokyo, Japan). Cells were maintained in RPMI-1640 supplemented with 10% fetal bovine serum (FBS), 2 mM L-glutamine, 100 units $ml^{-1}$ penicillin and 100 μg $ml^{-1}$ streptomycin sulphate.

### In vitro invasion assay

Invasion assay was performed by a modification of the methods of Albini et al (1987). Artificial basement membranes of a Transwell cell culture chamber (Costar Corp., Cambridge, MA, USA) were prepared by coating 6.5-mm-diameter polycarbonate filters (8-μm pore size, polyvinylpyrrolidone-free) with 25 and 150 μg of type I collagen obtained from rat tail tendon collagen (Collaborative Biomedical Corp., Bedford, MA, USA). The lower compartments of blind-well invasion chambers were filled with 700 μl RPMI-1640 supplemented with 10% FBS. Cells ($1 \times 10^5$) suspended in a total volume of 200 μl of invasion medium were then added to the upper invasion chamber compartments. After 8 and 24 h of incubation in a tissue culture incubator, the filters were fixed with 70% ethanol and stained with Giemsa solution. The number of cells that migrated through the membrane were counted in six random microscope fields of × 200 magnification of the lower filter surface.

### Northern blot analysis

Twenty micrograms of total RNA per lane was electrophoretically separated on a 1.0% agarose gel. After transfer the nylon membrane (Hybond™-$N^+$, Amersham Corp., Tokyo, Japan) was prehybridized for 45 min at 65°C, hybridized for 90 min with a $^{32}P$-random primer-labelled probe using a hybridization solution (Amersham Corp., Tokyo, Japan) and washed to a final stringency of 65°C in 1 × standard saline citrate (SSC) and 0.1% sodium dodecyl citrate (SDS). The syndecan-1 probe was a 490 bp *Eco*RI fragment of mouse syndecan-1 cDNA (pMB-29, kindly provided by Dr M Bernfield) and the syndecan-4 probe was a 370 bp *Eco*RI and *Bam*HI fragment of human syndecan-4 cDNA (pMB-237, kindly provided by Dr M Bernfield). Ribosomal RNA expression was used as an internal control.

### Vector construction and transfection

PR-39 fragment (nucleotide 4–539) containing a whole coding region of pig PR-39 gene (Storici and Zanetti, 1993) was amplified from pig small intestine mRNAs by RT-PCR (reverse transcriptase-polymerase chain reaction) using a primer (5′-GGGCTCAGGATTCACCAAAAGCTTTTAATGGGT-3′) for RT and two primers (5′-CTCACCTGGGCACCATGGAG-3′, 5′-GGGTATGTTATCAGCCACTC-3′) for PCR, and ligated into pCR™II vector (Amersham Corp., Tokyo, Japan). The PR-39 fragment in pCR™II vector was excised with *Hin*dIII–*Xba*I, and ligated into pRc/CMV expression vector (Invitrogen, San Diego, CA, USA). Syndecan-1 fragment (nucleotide 92–1289) containing a whole coding region of mouse syndecan-1 gene was amplified from clone pMB-5 (kindly provided by Dr M Bernfield) (Saunders et al, 1989) by PCR using two primers (5′-AGTAAGCTTACAGCCCTCGCTCGA-3′, 5′-CAATGTCTAGAGGACATGACCCAG-3′) and ligated into pCR™II vector. The fragment was excised with *Hin*dIII–*Xba*I, and ligated into pRc/CMV expression vector. Human HCC cells, HLF, expressing a low level of syndecan-1 (Matsumoto et al, 1997), were transfected with three plasmids, pRc/CMV vector containing pig PR-39, mouse syndecan-1 or no insert, respectively. For transfections, 40 μg of purified plasmid and 30 μg of Lipofectin (Gibco-BRL, Gaithersburg, MD, USA) were added to HLF cells (approximately 70%) in 100-mm culture dishes. After approximately 3 weeks, selection with geneticin (1000 μg $ml^{-1}$), viable colonies were subcultured using cloning rings.

### Immunoprecipitation and Western blot of PR-39

Cells were lysed in 25 mM Tris–HCl, pH 7.5, 150 mM sodium chloride (TBS) plus 0.5% Nonidet P-40 and PR-39 was immunoprecipitated from 1 mg of the total cell protein or 20 ml culture medium with anti-PR-39 polyclonal antiserum, which was produced by immunization of rabbit with 39 amino acids oligopeptide of PR-39 in our laboratory. After washing three times with TBS containing 0.25% Tween-20, the antibody–antigen complexes were dissociated by heating at 100°C for 4 min in standard Laemmli sample buffer, and the material separated on a 4–20% Tris–glycine gradient polyacrylamide gel (Bio-Rad Laboratories, Hercules, CA, USA). Gels were electroblotted onto nitrocellulose membranes (Bio-Rad Laboratories), incubated with anti-PR-39 antiserum, horseradish-conjugated goat anti-rabbit IgG (Seikagaku Kogyo, Tokyo, Japan), and the immune complexes were detected by enhanced chemiluminescence (ECL) detection system (Amersham International plc, Buckinghamshire, UK).

### Western blot of syndecan-1

The extracellular domain of syndecan-1 was isolated from cells as previously reported with slight modification (Koda et al, 1985). The cell pellets were resuspended with 20 mM Tris-buffered saline pH 7.4 containing 5 mM EDTA and sonicated. To release the extracellular domain of syndecan-1, the whole cell extract after sonication was placed on ice for 10 min after an addition of 20 μg $ml^{-1}$ bovine pancreatic trypsin (type III, Sigma Chemical Co, St Louis, MO, USA). The reaction was stopped by adding 100 μg $ml^{-1}$ soybean trypsin inhibitor (type I-S, Sigma Chemical Co, St Louis, MO, USA). The extracellular domain was partially purified by DEAE Sephacel (Pharmacia Biotech, Piscataway, NJ, USA) as previously reported (Koda et al, 1985). To prepare the core protein of the extracellular domain of syndecan-1, GAG side-chains were removed by treatment with heparin–sulphate lyase (heparitinase, EC 4.2.2.8, Seikagaku Kogyo, Tokyo, Japan) and chondroitin ABC lyase (chondroitinase ABC, EC 4.2.2.4. Seikagaku Kogyo, Tokyo, Japan) (Rapraeger et al, 1985). The extracellular domain of syndecan-1 before removal of GAG side-chains was separated on

 © 1999 Cancer Research Campaign

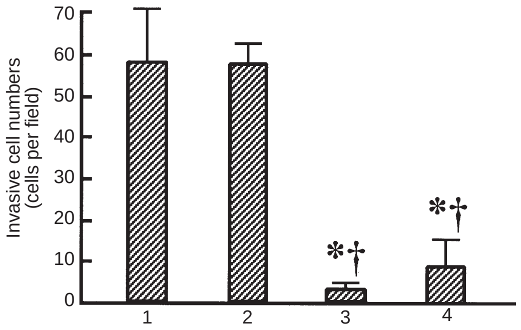

**Figure 1** Invasive activity of human hepatocellular carcinoma cells. Invasive activity was assayed in vitro by type I collagen (25 μg) coated 8-μm pore size filter of the invasion chamber. The number of cells that invade the filters after 24 h was counted in six random microscope fields of × 200 magnification of the lower filter surface to represent the invasive activity. Invasive activity of both HLE (lane 1) and HLF (lane 2) cells was significantly higher than that of HuH-1 (lane 3) and HuH-2 (lane 4). *Values for invasive activities significantly different from HLE (lane 1) ($P < 0.001$). †Values for invasive activities significantly different from HLF (lane 2) ($P < 0.001$)

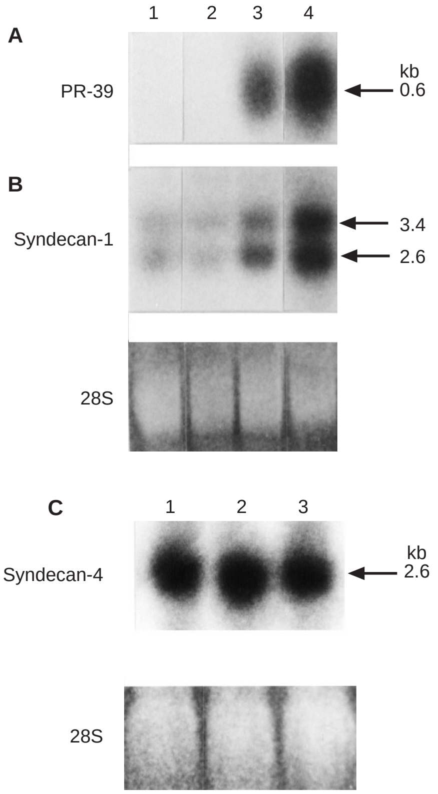

**Figure 2** Expression of transfected PR-39 and endogenous syndecan-1 and syndecan-4 genes examined by Northern blot. (**A**) Northern blot analysis using pig PR-39 cDNA probe revealed the strong hybridization signals for pig PR-39 in two PR-39 transfectants (lanes 3 and 4) at the size of approximately 0.6 kb, but no signals were observed in parental HLF cells (lane 1) or a vector only transfectant (lane 2). (**B**) Northern blot analysis using human syndecan-1 cDNA probe revealed the induction of the endogenous syndecan-1 gene detected at the sizes of 3.4 and 2.6 kb in the two PR-39 transfectants (lanes 3 and 4) compared with the controls (lanes 1 and 2). Ribosomal RNA was used for loading controls. Lane 1: HLF cells, lane 2: a vector-only transfectant, lanes 3 and 4: PR-39 transfectants (P1 and P2). (**C**) Northern blot analysis using human syndecan-4 cDNA probe revealed syndecan-4 gene expression detected at 2.6 kb was not affected by PR-39 gene transduction. Syndecan-4 was expressed highly both in PR-39 transfectants (lanes 2 and 3) and HLF cells (lane 1). Ribosomal RNA was used for loading control. Lane 1: HLF cells, lanes 2 and 3: PR-39 transfectants (P1 and P2)

5% polyacrylamide gel and the core proteins after removal of GAG side-chains were separated on a 7% polyacrylamide gel containing 2.6 M urea using a running buffer composed of 40 mM Tris, 60 mM boric acid, 0.8 mM EDTA, 1 mM $Na_2SO_4$, 0.1% SDS, and electrophoretically transferred to Immobilon N membrane (Millipore, Bedford, MA, USA) as previously reported (Rapraeger et al, 1985). The membrane was blocked with 5% dry milk, 10 mM Tris-HCl pH 7.4, 0.15 M sodium chloride, 0.05% sodium azide for 30 min at room temperature, incubated with anti-mouse syndecan-1 antibody, 281–2 (PharMingen, San Diego, CA, USA) at 4°C overnight, and washed with 20 mM Tris–HCl pH 7.4, 0.15 M sodium chloride, 0.05% Tween-20 (TBS/Tween). The membrane was incubated for 1 h at room temperature with horseradish peroxidase conjugated anti-rabbit IgG, washed with TBS/Tween and visualized with ECL detection system.

### Phagokinetic track motility assay

Uniform carpets of gold particles were prepared on coverslips coated with bovine serum albumin or type I collagen (1 mg $ml^{-1}$), as described by Albrecht-Buehler (1977). Colloidal gold-coated coverslips were placed in 35-mm tissue culture dishes containing 2 ml culture medium with 10% FBS, and then 2000 cells were added to each plate. After 48 h, phagokinetic tracks were visualized using dark-field illumination in an Olympus inverted microscope at a magnification of 200 ×. The area cleared of gold particles by at least ten cells selected randomly was measured. Furthermore, in order to examine the relative contribution of syndecan-1 to inhibit motility on Type I collagen-coated plates, cells were grown and assayed in the presence of 30 mM sodium chlorate, which at this dose completely prevents the sulphation of glycosaminoglycan.

### Immunofluorescent analysis of syndecan-1

Cells grown on coverslips were fixed with freshly prepared 2% paraformaldehyde in phosphate-buffered saline (PBS) for 20 min and incubated anti-mouse syndecan-1 antibody, 281-2, or anti-human syndecan-1 antibody, BB4 (Serotec Ltd, Oxford, UK) for 1 h. After washing with PBS, the cells were incubated with fluorescent Cy3-conjugated rabbit anti-rat IgG or goat anti-mouse IgG (Biological Detection Systems Inc., Pittsburg, PA, USA), mounted on glass slides with PermaFluor Aqueous Mountant (Lipshaw Immunon, Pittsburg, PA, USA), and visualized using Axioscope FL microscopy (Carl Zeiss Inc., Jena, German).

### Staining of actin filament

Cells grown on coverslips were rinsed twice with a cytoskeletal stabilizing buffer (4 M glycerol, 25 mM PIPES [pH 6.9], 1 mM EGTA and 1 mM magnesium chloride), incubated in the same buffer containing 0.2% Triton X-100 for 5 min at 20°C to extract soluble proteins, and fixed for 45 min at 20°C in 3.7% formaldehyde in PBS. After two 10-min washes in PBS, cells were incubated with rhodamine-conjugated phalloidin (Amersham Corp., Tokyo, Japan) diluted 1:50 in PBS containing 0.5 mg $ml^{-1}$ bovine serum albumin for 30 min. Cells were mounted with PermaFluor Aqueous Mountant, and observed with Laser Scanning Confocal Imaging System MRC-600 (Bio-Rad, Hertfordshire, UK).

© 1999 Cancer Research Campaign

### Statistical analysis

Student's *t*-test or Welch's *t*-test was used for statistical analysis of invasion and motility assays.

### RESULTS

### Invasive activity of four human HCC cell lines

We have previously reported about mRNA and protein expressions of syndecan-1 in the four human HCC cell lines (HLE, HLF, HuH-1 and HuH-2); the expressions of syndecan-1 in HLE and HLF cells were very low compared with those of HuH-1 and HuH-2 cells at both mRNA and protein levels (Matsumoto et al, 1997). In order to select an appropriate cell line, which showed a low expression of syndecan-1 and a high invasive activity for the experiment of PR-39 gene transfection, we examined the invasive activities of these cell lines. A cell number that passed through type I collagen (25 µg)-coated filter of the invasion chamber after 24 h was represented as in vitro invasive activity. We used type I collagen rather than conventional matrigel system as an extracellular matrix model because approximately 80% of human HCCs develop from cirrhotic livers, in which 70% of total collagens were type I (Aycock and Seyer, 1989), and syndecan-1 has a high binding affinity to type I procollagen, but less affinity to matrigel components such as laminin and type IV collagen (Koda et al, 1985; Elenius et al, 1990). Invasive activities of HLE and HLF cells (lanes 1 and 2, Figure 1) were significantly higher than those of HuH-1 and HuH-2 cells (lanes 3 and 4, Figure 1). Taking our previous results of mRNA and protein expression of syndecan-1 with the present results of invasion assay, it was concluded that the expression of syndecan-1 was inversely associated with the invasive activity in human hepatocellular carcinoma cells and so, therefore, HLF cell was an appropriate cell line to use for the present experiment because of an endogenously low syndecan-1 expression and a high invasive activity.

### Transfection with PR-39 gene into HLF cells and induction of syndecan-1 mRNA, but not of syndecan-4 mRNA

The cDNA coding for full length of precursor (pre-pro) form of pig PR-39 was transfected into HLF cells. Two PR-39 transfected clones, P1 and P2 (lanes 3 and 4, Figure 2A) were obtained after geneticin selection and showed a strong hybridization signal at 0.6 kb, the expected size for precursor PR-39 mRNA. In addition, P1 and P2 transfectants revealed the augmentation of two spliced forms of human syndecan-1 mRNA detected at 3.4 and 2.6 kb (lanes 3 and 4, Figure 2B) as compared with the parental HLF and the vector only transfectant (lanes 1 and 2, Figure 2A). On the other hand, syndecan-4 mRNA expression was not affected by PR-39 gene transduction; syndecan-4 was expressed highly both in PR-39 transfectants (lanes 2 and 3, Figure 2C) and HLF cells (lane 1, Figure 2C). These data indicate that the PR-39 gene transduction to HLF cells augmented endogenous syndecan-1 mRNA but not syndecan-4 mRNA, confirming our assumption that PR-39 gene transduction worked as a syndecan-1 inducer.

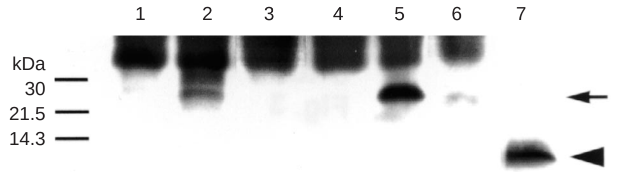

**Figure 3** PR-39 gene product in cytoplasm and culture medium of PR-39 transfectants. Immunoreactive bands of an approximately 27 kDa, a precursor form of PR-39 (arrow), were obtained both in the cytoplasm (lane 2) and in the culture medium (lane 5). These bands were almost diminished by antisera pre-absorbed with 100 µg of synthetic PR-39 peptide (lane 3 and lane 6). Synthetic PR-39 peptide was detected at approximately 5 kDa (lane 7), but a mature form of PR-39 could not be detected in this condition (arrowhead). Lane 1: cytoplasm of HLF cells, lanes 2 and 3: cytoplasm of PR-39 transfectant (P2), lane 4: medium of HLF cells, lanes 5 and 6: medium of PR-39 transfectant (P2), lanes 3 and 6: antisera pre-absorbed with synthetic PR-39 peptide, lane 7: synthetic PR-39 peptide

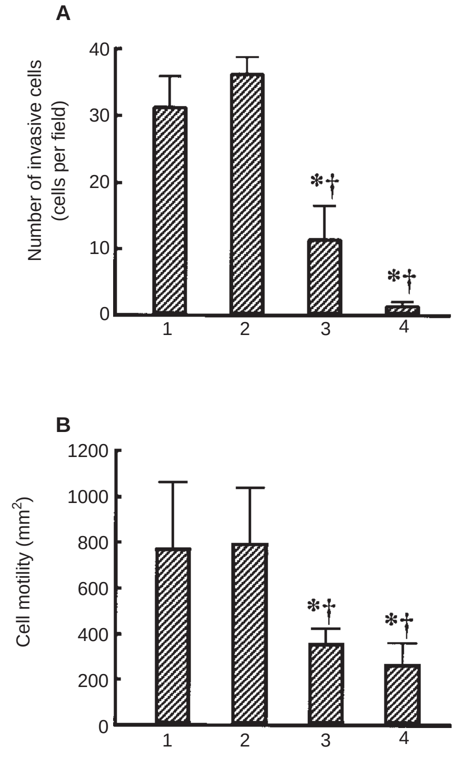

**Figure 4** Invasive activity into type I collagen and motile activity assayed by phagokinetic tracks of PR-39 transfectants. (**A**) Invasive activity was assayed in vitro by type I collagen (150 µg)-coated 8-µm pore size filter of the invasion chamber. A number of cells that invaded the filters after 8 h was counted in six random microscope fields of ×200 magnification of the lower filter surface to represent the invasive activity. Invasive activity of the two PR-39 transfectants (lanes 3 and 4) was significantly lower than that of the controls (lanes 1 and 2). Lane 1: HLF cells, lane 2: a vector-only transfectant, lanes 3 and 4: PR-39 transfectants (P1 and P2). *Values for invasive activities significantly different from HLF (lane 1) ($P < 0.001$). †Values for invasive activities significantly different from the vector-only transfectant (lane 2) ($P < 0.001$). (**B**) Motile activity of PR-39 transfectants was assayed by phagokinetic tracks. After 48 h, phagokinetic tracks were visualized using dark-field illumination in an inverted microscope at a magnification of 200×. The area cleared of gold particles by cells represented motile activity and was measured. The two PR-39 transfectants showed significant reduction of motile activity (lanes 3 and 4) compared with that of the controls (lane 1 and lane 2). Lane 1: HLF cells, lane 2: a vector-only transfectant, lanes 3 and 4: PR-39 transfectants (P1 and P2). *Values for motile activities significantly different from HLF (lane 1) ($P < 0.001$). †Values for motile activities significantly different from the vector-only transfectant (lane 2) ($P < 0.001$).

© 1999 Cancer Research Campaign

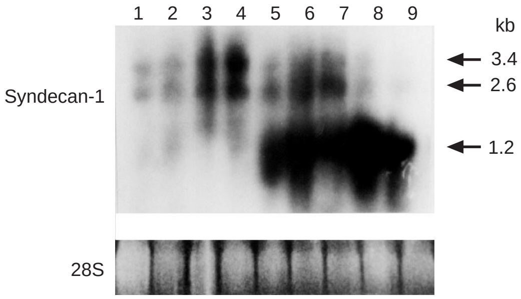

**Figure 5** Expression of transfected mouse syndecan-1 gene examined by Northern blot. Northern blot analysis using mouse syndecan-1 cDNA probe revealed the strong hybridization signals in the five syndecan-1 transfectants (lanes 5–9) at the size of approximately 1.2 kb in addition to the signals of endogenous human syndecan-1 at the sizes of 3.4 and 2.6 kb as seen in the parental cells and vector only transfectant (lanes 1 and 2). The signals of transfected syndecan-1 gene at 1.2 kb were stronger than those of augmented endogenous human syndecan-1 gene in PR-39 transfectants (lanes 3 and 4). Ribosomal RNA was used for loading control. Lane 1: HLF cells, lane 2: vector-only transfectant, lanes 3 and 4: PR-39 transfectants (P1 and P2), lanes 5–9: syndecan-1 transfectants (S1–S5)

### PR-39 gene product in cytoplasm and culture medium of PR-39 transfectants

To examine whether the transfectants produce PR-39 protein, Western blot analysis was performed after immunoprecipitation using anti-PR-39 antiserum (Figure 3). Immunoreactive bands of an approximately 27 kDa, a precursor form of PR-39, were obtained both in the cytoplasm (lane 2, Figure 3) and in the culture medium (lane 5, Figure 3). These bands were almost diminished by antisera pre-absorbed with 100 μg of synthetic PR-39 peptide (lanes 3 and 6, Figure 3). The bands were more intense in culture medium than in cytoplasm, suggesting that PR-39 is mainly secreted as a precursor form. A mature form of PR-39, of which molecular weight is approximately 5 kDa, could not be detected in this condition.

### Alteration of invasive and motile activities in PR-39 transfectants

To demonstrate the effect of PR-39 gene transduction on the invasive character of HLF cells, an assay using a type I collagen (150 μg)-coated filter of invasion chamber was performed. After 8 h of incubation, the cell number that had invaded the filter was counted in two transfectants, P1 and P2, parental cell and vector-only transfectant, respectively. P1 and P2 transfectants (lanes 3 and 4, Figure 4A) showed a significant decrease in invasive cell number, compared with those obtained in the parental HLF or the vector-only transfectant (lanes 1 and 2, Figure 4a). The results of 24-h incubation were similar to those of 8-h incubation (data not shown).

As such a change of invasive activities might be caused by the difference of motile activities in addition to cell–matrix interaction between tumour cells and type I collagen, we then performed a phagokinetic track assay. The area cleared of gold particles after 48 h was measured (Figure 4B). P1 and P2 transfectants (lanes 3 and 4, Figure 4B) showed significant reductions in motile activity compared with the parental HLF cells (lane 1, Figure 4B) or the vector-only transfected clone (lane 2, Figure 4B). These data indicate that PR-39 gene transduction reduces cell motility not simply through the cell-to-matrix interaction through the augmentation of syndecan-1 induction.

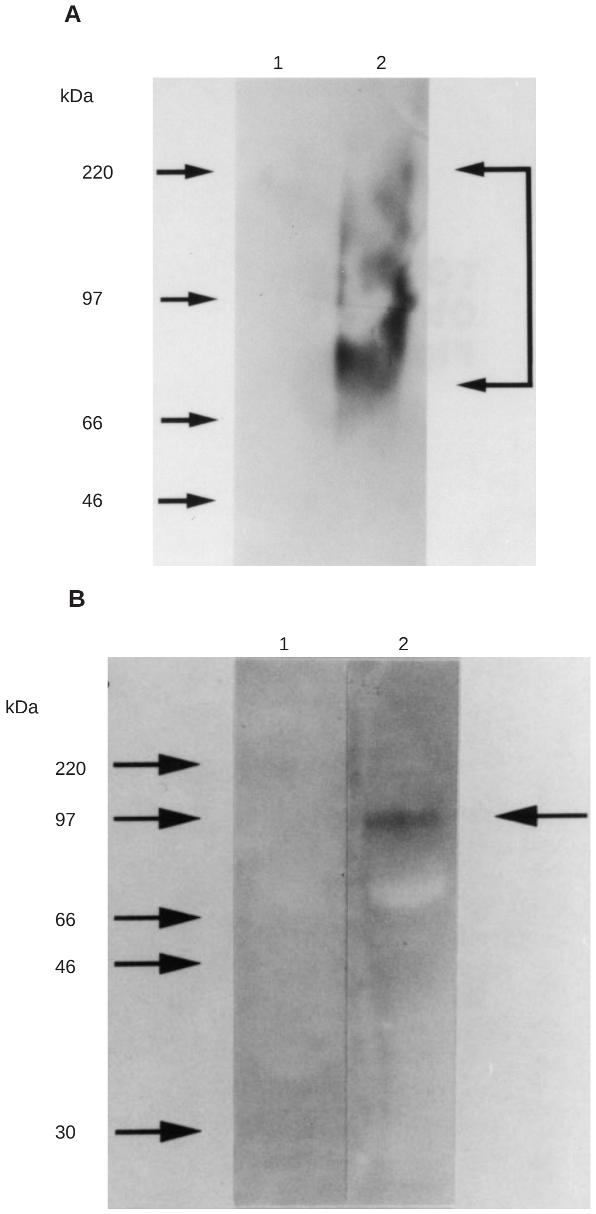

**Figure 6** Western blot of mouse syndecan-1 before and after removal of heparan sulphate chains. (**A**) The protein samples were separated on 5% SDS-polyacrylamide gel without removal of GAG chains. The 281–2 antibody recognized syndecan-1 protein as a smear. (**B**) The protein samples were separated on 7% SDS-polyacrylamide gel with removal of GAG chains by treatment with heparitinase and chondroitinase. The 281–2 antibody recognized an approximately 65 kDa core protein. Lane 1: HLF, lane 2: syndecan-1 transfectant (S2)

### Transfection with syndecan-1 gene into HLF cells

In order to elucidate this, the cDNA coding for full length of murine syndecan-1 was used independently and we investigated whether syndecan-1 gene transfection caused the same phenomena as syndecan-1 inducer PR-39 gene. We used murine syndecan-1 gene for the transfection into the human hepatocellular carcinoma cells because: (i) we could distinguish the product of transfected syndecan-1 from endogenous syndecan-1 easily by using the antibody which specifically recognized murine syndecan-1; (ii) murine syndecan-1 has been reported to have the same function in

© 1999 Cancer Research Campaign

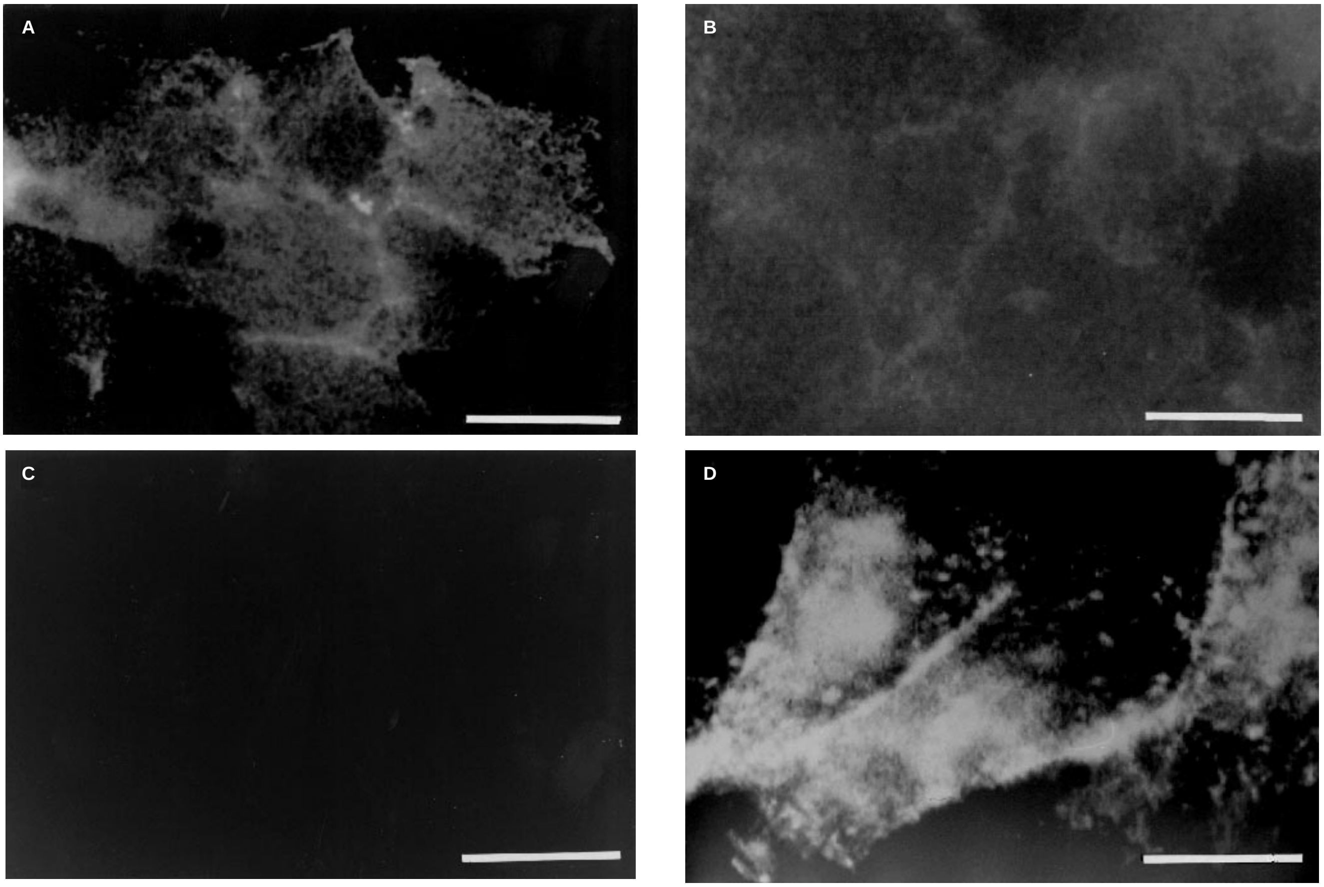

**Figure 7** Immunofluorescent analysis of syndecan-1 at cell surface in PR-39 or syndecan-1 transfectants. BB4 and 281–2 antibodies were used for human and mouse syndecan-1 respectively. Using BB4 antibody, the higher immunofluorescence was observed at the cell surface in PR-39 transfectant (**A**) than that of syndecan-1 transfectant (**B**). However, syndecan-1 gene transfectant showed a markedly high immunofluorescence at the cell surface using 281–2 antibody (**D**), while PR-39 transfectant showed no immunofluorescence (**C**). **A** and **C**: PR-39 transfectant (P2), **B** and **D**: syndecan-1 transfectant (S2), **A** and **B**: human syndecan-1, **C** and **D**: mouse syndecan-1. Scale bar, 15 µm human cells as in murine cells (Liebersbach and Sanderson, 1994). Five syndecan-1 gene transfected clones, S1–S5 (lanes 5–9, Figure 5) were obtained after selection by geneticin and showed the strong hybridization signals for mouse syndecan-1 mRNA at a size of approximately 1.2 kb, which was consistent with the expected size of the polyadenylated product of the mouse syndecan-1 expression vector, while the weak endogenous signals of human syndecan-1 mRNA at 3.4 and 2.6 kb, as seen in the parental HLF cells (lane 1, Figure 5) and vector-only transfected cells (lane 2, Figure 5), were also obtained. The mRNA levels of transfected mouse syndecan-1 gene at 1.2 kb (lanes 5–9, Figure 5) were much higher than those of augmented human syndecan-1 gene in PR-39 transfectants (lanes 3 and 4, Figure 5).

To demonstrate that the syndecan-1 transfectants produced syndecan-1 with heparan sulphate chains, we performed Western blots of mouse syndecan-1 before and after removal of heparan sulphate chains. The protein samples were separated on 5% SDS-polyacrylamide gel without any treatments and then on 7% SDS-polyacrylamide gel with removal of GAG chains treated with heparitinase and chondroitinase. The 281–2 antibody recognized the syndecan-1 protein as a smear without treatment (lane 2, Figure 6A). Following removal of GAG chains from the protein, the antibody specifically recognized a 65 kDa core protein in syndecan-1 transfectants (lane 2, Figure 6B). These data indicate that the syndecan-1 transfectants produced syndecan-1 with heparan sulphate chains.

### Syndecan-1 expression at cell surface in PR-39 or syndecan-1 transfectants

To show levels of syndecan-1 at the cell surface, we performed immunofluorescent analysis using an anti-human syndecan-1 antibody (BB4) which recognizes human syndecan-1 specifically, or an anti-mouse syndecan-1 antibody (281–2) which recognizes mouse syndecan-1 specifically. Using BB4 antibody, we observed the higher immunofluorescence at the cell surface in PR-39 transfectants (Figure 7A) than those of parental HLF, vector-only transfectant and syndecan-1 transfectants (Figure 7B is a representative of these three cell lines). However, syndecan-1 gene transfectants showed a markedly high immunofluorescence at the cell surface using 281–2 antibody (Figure 7D), while parental HLF, Mock and PR-39 transfectants showed no immunofluorescence (Figure 7C is a representative of these three cell lines).

### Alteration of invasive activity, but not motile activity in syndecan-1 transfectants

By using these transfectants, the invasive activity of the control HLF cells, the vector-only transfected clone and the five

© 1999 Cancer Research Campaign

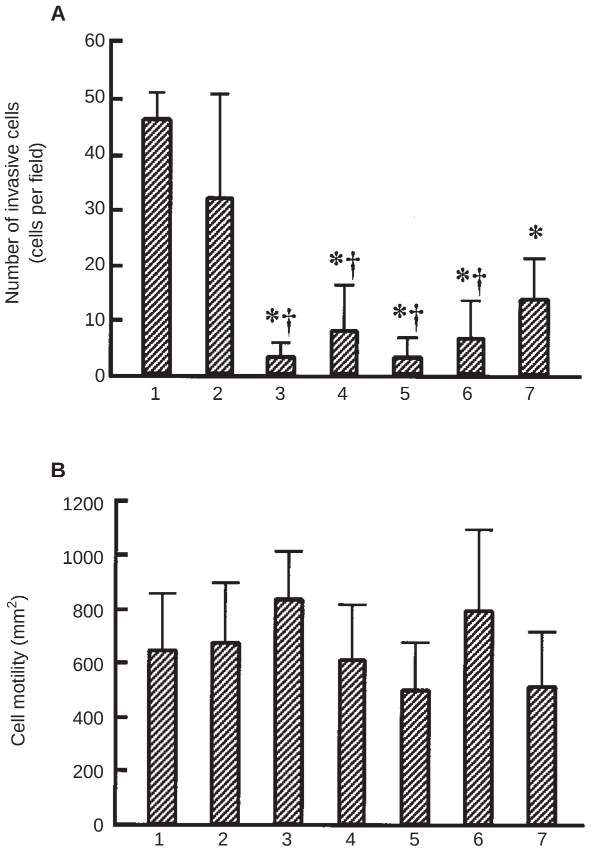

**Figure 8** Invasive activity into type I collagen and motile activity assayed by phagokinetic tracks of syndecan-1 transfectants. (**A**) Invasive activity was assayed in vitro by type I collagen (150 μg)-coated 8-μm pore size filter of the invasion chamber. The number of cells that invaded the filters after 24 h was counted in six random microscope fields of ×200 magnification of the lower filter surface to represent the invasive activity. All of the five syndecan-1 transfectants (lanes 3–7) showed significant reduction of invasive activity compared with that of the HLF cells (lane 1) and the vector only transfectant (lane 2). Lane 1: HLF cells, lane 2: a vector only transfectant, lanes 3–7: syndecan-1 transfectants (S1–S5). *Values for invasive activities significantly different from HLF (lane 1) ($P < 0.001$). †Values for invasive activities significantly different from the vector-only transfectant (lane 2) ($P < 0.05$). (**B**) Motile activity of syndecan-1 transfectants was assayed by phagokinetic tracks. After 48 h, phagokinetic tracks were visualized using dark-field illumination in an inverted microscope at a magnification of 200×. The area cleared of gold particles by cells represented motile activity and was measured. Virtually no difference was observed among the five syndecan-1 transfectants (lanes 3–7) and the controls (lanes 1 and 2). Lane 1: HLF cells, lane 2: a vector-only transfectant, lanes 3–7: syndecan-1 transfectants (S1–S5)

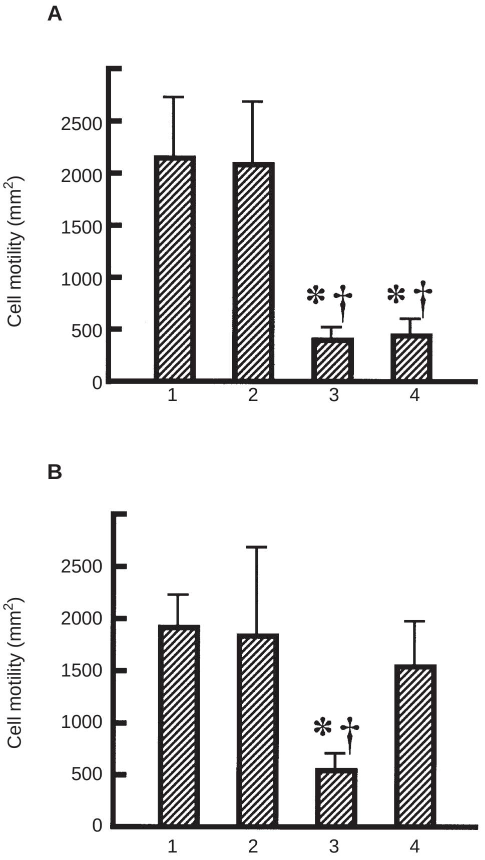

**Figure 9** Motility assay on type I collagen-coated plates with or without chlorate treatment. (**A**) Both of PR-39 and syndecan-1 transfectants showed significant reductions of motile activity on type I collagen-coated plates without chlorate (lanes 3 and 4) compared with parental HLF and vector-only transfectants (lanes 1 and 2). (**B**) PR-39 transfectants treated with chlorate showed significant reductions of motile activity on type I collagen-coated plates (lane 3), while syndecan-1 transfectants treated with chlorate showed no significant reductions (lane 4). *Values for motile activities significantly different from HLF ($P < 0.001$). †Values for motile activities significantly different from the vector-only transfectant ($P < 0.001$). Lane 1: HLF, lane 2: vector-only transfectant, lane 3: PR-39 transfectant (P2), lane 4: syndecan-1 transfectant (S2)

syndecan-1 transfected clones were all assayed by the same procedure used in the experiment for PR-39 gene transfectants. In the experiment, the number of cells that invaded the filters was counted after 24 h, as at 8 h, the interval used in PR-39 experiment, the preliminary experiments had shown insufficient differentiation of invasive activity between the syndecan-1 transfectants and the control cells. All of the five syndecan-1 transfectants, S1–S5 (lanes 3–7 in Figure 8A) showed a significant reduction of invasive activity compared with that of the parental HLF cells and the vector-only transfectant cells (lanes 1 and 2, Figure 8A). Motile activity of the HLF cells, the vector-only transfected clone and the five syndecan-1 transfectants was assayed by phagokinetic tracks under the same conditions used in the PR-39 transfectants. However, the S1–S5 clones (lanes 3–7, Figure 8B) showed no difference of motile activity compared with the parental HLF cells or the vector-only transfected clone (lanes 1 and 2, Figure 8B).

### Effect of chlorate treatment on motile activity of PR-39 transfectants and syndecan-1 transfectants

Furthermore, we performed motility assay on type I collagen-coated plates with or without chlorate treatment which prevents the sulphation of glycosaminoglycan. Both of PR-39 and syndecan-1 transfectants showed significant reductions of motile activity on type I collagen-coated plates (lanes 3 and 4, Figure 9A) compared with parental HLF and vector-only transfectants (lanes 1 and 2, Figure 9A). PR-39 transfectants treated with chlorate still showed significant reductions of motile activity on type I collagen-coated plates (lane 3, Figure 9B), while syndecan-1 transfectants treated with chlorate almost returned to the same motile activity as that of parental cells (lane 4, Figure 9B). Therefore, our results convinced us that the role of PR-39 gene transduction in suppressing tumour invasion is not restricted to the augmentation of syndecan-1 expression.

*© 1999 Cancer Research Campaign*

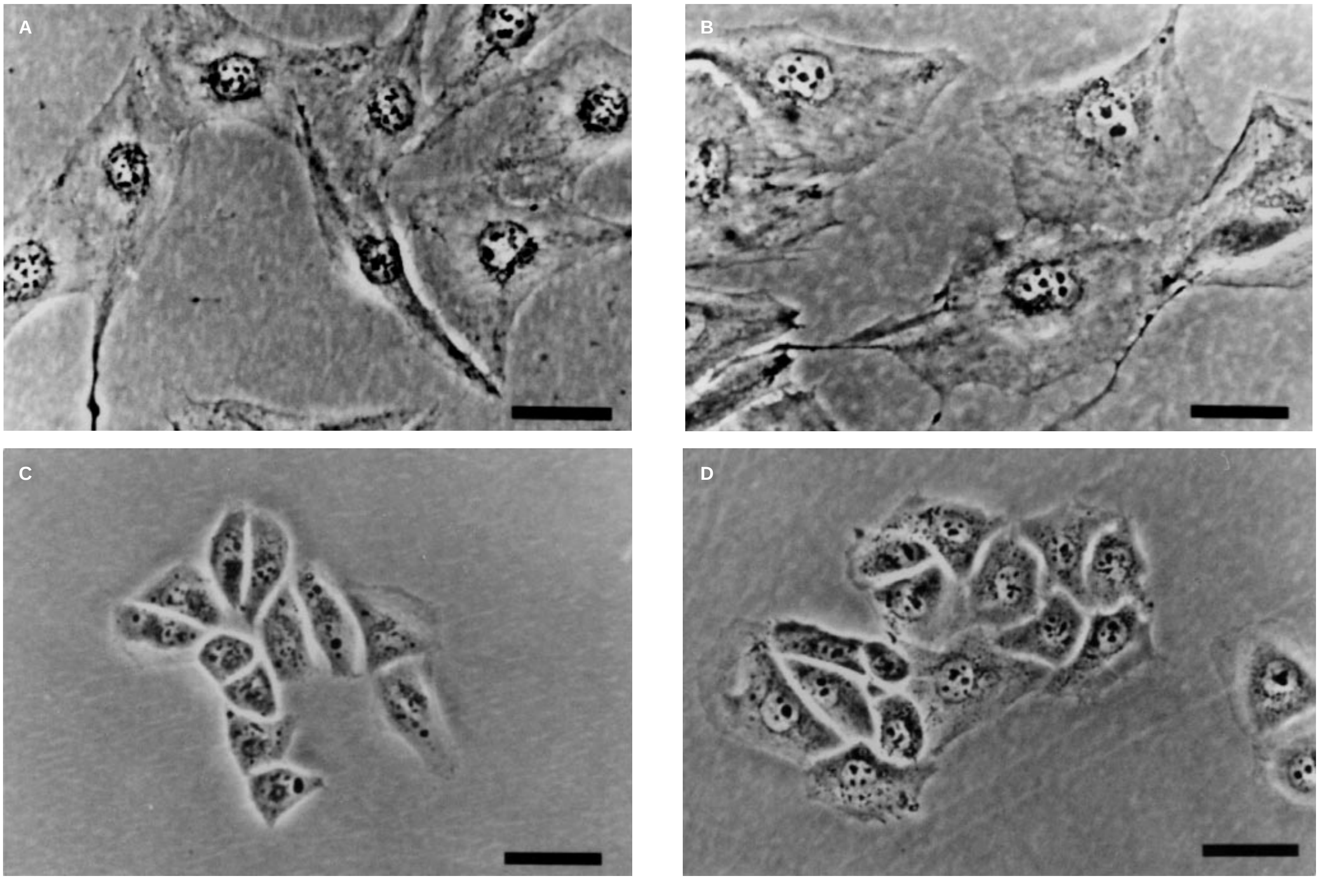

**Figure 10** Cell morphology of PR-39 transfectants. Morphology of the parental HLF cells (**A**) and a vector-only transfectant (**B**) examined by phase-contrast microscopy was a flat shape with short or long spikes, but PR-39 transfectants, P1 and P2, revealed a more compact shape without the spikes (**C** and **D**). Scale bar, 15 μm

### Alteration of morphology and actin structure of PR-39 transfectant cells

As the motile activity of PR-39 transfected cells was reduced, we next examined the morphology and actin structures of the cells because of their bearing on cell motility. The phase-contrast microscopic examinations of the parental HLF cells and the vector-only transfectant revealed a flat form with short or long spikes (Figure 10 A, B). On the other hand, PR-39 transfectants, P1 and P2 formed more compact cells without spikes (Figure 10 C, D), indicating that PR-39 gene transfection clearly influences the cell morphology. Furthermore, actin filaments were stained with rhodamine-conjugated phalloidin and actin structures were observed with Laser Scanning Confocal Imaging System. The parental HLF cells and the vector-only transfectant revealed intense staining of fine microfilament bundles oriented parallel to the long axes of the cells (Figure 11 A, B), whereas P1 and P2 revealed thin, short microfilament bundles or disorganization of the microfilaments (Figure 11 C, D).

In contrast, syndecan-1 transfectants showed a flat form with short or long spikes, which was indistinguishable from both the parental HLF cells and the vector-only transfectant examined by phase-contrast microscopy (data not shown). The actin structure of S1–S5 clones revealed intense staining of fine microfilament bundles oriented parallel to the long axes of the cells, which was indistinguishable from that of both the parental HLF cells and the vector-only transfectant (data not shown). Therefore, alteration of cell morphology and actin structure might be important for the suppression of motile activity in PR-39 transfectants; this would be a new function of PR-39 in addition to its augmentation of syndecan-1.

### DISCUSSION

In this study, we obtained three findings:

1. PR-39 gene transduction to human hepatocellular carcinoma cells induced syndecan-1 expression.
2. PR-39 gene transduction suppressed invasive and motile activities, while syndecan-1 gene transduction suppressed invasive activity but not motile activity.
3. PR-39 gene transduction altered cell morphology and actin structure, but syndecan-1 gene transduction did not.

Previously, we found that the expression of syndecan-1 was reduced in human HCC with high metastatic potential and speculated that syndecan-1 played an important role in the inhibition of invasion and metastasis (Matsumoto et al, 1997). Accordingly, it is assumed that the modification of this process by inducing syndecan-1 may become a new strategy to inhibit these processes. In 1994, Gallo et al attempted to identify soluble extracellular factors that induce syndecan-1 expression from a pig skin wound fluid, because the cutaneous wound environment is a potential

© 1999 Cancer Research Campaign

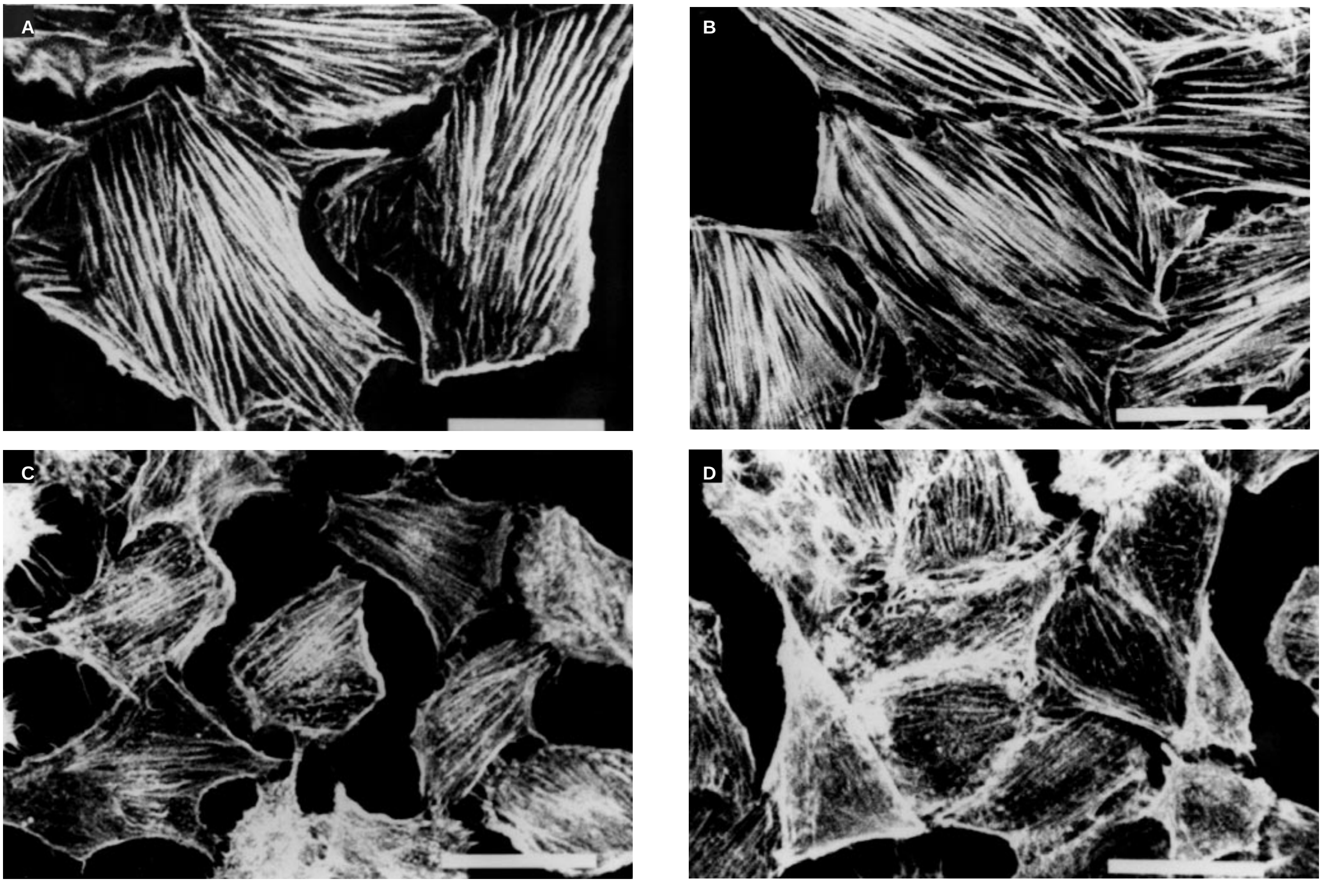

**Figure 11** Actin structure of PR-39 transfectants. Actin structure was examined by staining of actin filaments using rhodamine-conjugated phalloidin. The HLF cells (**A**) and a vector-only transfectant (**B**) revealed an intense staining of fine microfilament bundles oriented parallel to the long axes of the cells, whereas P1 and P2 revealed thin, short microfilament bundles or a disorganization of the microfilaments (**C** and **D**). Scale bar, 15 μm source of inductive factors (Gallo et al, 1994). They isolated a 4.7-kDa protein that induced syndecan-1 in mouse NIH 3T3 fibroblasts and found that it was identical to PR-39 by amino acid sequence analysis. In a preliminary experiment, we found that the synthetic peptide of PR-39 augmented syndecan-1 mRNA expression in HLF cells in addition to in NIH 3T3 cells, although the amount required for syndecan-1 induction was more than 10 mg ml$^{-1}$, being rather high (unpublished observation). Because the available amount of synthetic peptide was limited and pI of the peptide was very basic due to the high content of proline and arginine, and was easy to adhere non-specifically, we attempted to transfect the PR-39 gene into HCC cells in order to obtain the continuous expression of PR-39 in the cells. Two transfectants with PR-39 gene, which were selected by geneticin and were stably expressing PR-39, showed the induction of syndecan-1 mRNA but not syndecan-4 (Figure 2). The induction of syndecan-1 at the cellular level in PR-39 transfectants was also confirmed by immunofluorescent analysis using anti-human syndecan-1 antibody (Figure 7C).

These results support our assumption that PR-39 gene transduction works as a syndecan-1 inducer in HLF cells. An important issue is what kind of mechanism is involved in the syndecan-1 induction by PR-39 gene transduction. It is reported that a biologically active segment of PR-39 is C-terminal proline-rich region. However, our Western blot analysis mainly showed a precursor form of PR-39 both in the cytoplasm and in the culture medium (Figure 3), suggesting that PR-39 is mainly secreted as a precursor form, not a clipped mature region of PR-39. Usually, mature PR-39 arises from the interaction with the serine protease of azurophilic granules in neutrophils (Boman, 1995). Therefore the reason the product of HLF cells overexpressing PR-39 gene is a precursor form may be explained by the fact that HLF cells lack neutrophil azulophilic granules and thus the precursor form is secreted extracellularly without processing. However, it is noteworthy that the PR-39 gene transduction works as a syndecan-1 inducer, which was confirmed by the results including endogenous syndecan-1 induction and various changes of cell motilities and morphologies. The possible explanations for this phenomenon are that immature PR-39 directly functions as mature PR-39 or a very small amount of mature PR-39 exists spontaneouly and functions, but can not be detected by Western blot. Concerning the function of PR-39, Chan and Gallo (1998) very recently postulated that extracellular PR-39 passed through cell membrane and binded intracellular binding proteins (receptor). According to their assumption, intracellular PR-39 or its precursor may also bind intracellular binding molecule without excreting outside the cell. Very recently, we found several intracellular proteins which interact with the chimeric protein composed of PR-39 precursor and haemagglutinin, which was made by transfection of chimaeric genes into HLF cells (not published). Further studies are needed to clarify this issue.

*© 1999 Cancer Research Campaign* 

The transfection experiments of the PR-39 gene and the syndecan-1 gene, both of which showed significant suppression of invasive activity in type I collagen-coated filter, demonstrated that the invasive activity of human HCC cells was modified by syndecan-1 expression. The fact that syndecan-1 gene transfection induced a functional syndecan-1 molecule was confirmed by SDS polyacrylamide gel electrophoresis with or without heparitinase treatment, by immunofluorescent analysis using anti-mouse syndecan-1 antibody and by motile assays treated with sodium chlorate, an inhibitor of sulphation of glycosaminoglycan. In human B lymphoid cell line, the transfection of syndecan-1 gene inhibited invasion into type I collagen by the cell binding (Liebersbach and Sanderson, 1994). This observation also supports one of our conclusions that cell-to-matrix interaction through syndecan-1 and type I collagen is required to inhibit tumour cell invasion. As pointed out by Liotta, the metastatic cascade consists of a series of steps which include: leaving the primary tumour, invading the local tissue, entering the circulation, arresting at the distant vascular bed, extravasating into the target organ interstitium and parenchyma, and proliferating as a secondary colony (Liotta et al, 1986, 1991). Among these steps, tumour cell invasion is one of the most important (Goyette et al, 1992; Dickson et al, 1996; Effert et al, 1996). Our results using the transfection of PR-39 gene to tumour cells showed that the induction of syndecan-1 by PR-39 could result in the inhibition of tumour cell invasion.

In addition to the function of PR-39 that can induce syndecan-1 expression, we found another effect of PR-39 gene transduction: PR-39 gene transfectants showed alterations of motile activity and actin structure not observed in syndecan-1 gene transfectants. Furthermore, the motile activities of PR-39 transfectants were not affected by chlorate treatment, suggesting strongly that PR-39 has more functions than syndecan-1 induction. Two possible mechanisms were evolved for the alteration of cell motility: one was an involvement of a special class of enzymes called motor proteins walking or sliding along a microfilament or a microtubule; the other was the changes of cell shape which resulted from the polymerization and assembly of actin (Stossel, 1993). The latter mechanism seems to be more likely in the present case, because alterations of motile activity and actin structure occurred simultaneously in the PR-39 gene transfectants. Thus, it is concluded that PR-39 functions as a modifier of motile activity and actin structure, possibly through association with the proteins which control actin polymerization and assembly of actin filaments, such as actin-cross-linking proteins, small actin-binding proteins, actin-severing proteins, actin-capping proteins or small GTP-binding proteins. Moreover, new functions of PR-39 have been reported that it inhibits phagocyte NADPH oxidase activity of pig neutrophils by binding to Src homology 3 (SH3) domains of $p47^{phox}$ (Shi et al, 1996) and that PR-39 binds and affects a SH3-containing signal transduction protein, P130Cas (Chan and Gallo, 1998). Therefore, we expect that PR-39 may bind to SH3 domain of various intracellular proteins based on two previous observations: (i) proline-rich motifs have an ability to bind to SH3 domains (Yu et al, 1994; Pawson, 1995); (ii) PR-39 has five repeats of the proline-rich motif, PXXPPXXP (Storici and Zanetti, 1993), whose prototype is Sos, an activator of the *ras* guanine nucleotide exchange protein (Gout et al, 1993; Musacchio et al, 1994). Thus, the alteration of actin structure caused by PR-39 gene transduction might result from the binding of the proline-rich region of PR-39 to SH3 domain of some protein associated with polymerization or assembly of actin filaments, or intracellular signalling. Further studies are needed to clarify this point.

It is also important to clarify how PR-39 induces syndecan-1 expression at mRNA level in HCC cells. A number of mechanisms of syndecan-1 synthesis have been reported in different tissues: a transcriptional regulation (Elenius et al, 1992), post-transcriptional regulation (Sanderson et al, 1992; Vainio and Thesleff, 1992; Yeaman and Rapraeger, 1993) and post-translational regulation (Sanderson and Bernfield, 1988). In NIH 3T3 cells, the combination of basic fibroblast growth factor (bFGF) and transforming growth factor β (TGF-β) at high concentrations increased syndecan-1 expression at a transcriptional level (Elenius et al, 1992). In our experiment, syndecan-1 was induced both at mRNA and protein levels, suggesting that a transcriptional or a post-transcriptional regulation should be considered. However, an alteration of mRNA stability of syndecan-1, which is a major cause of post-transcriptional regulation, has not been reported. To assess the transcriptional regulation of syndecan-1, the sequence upstream of the first exon of syndecan-1 gene was investigated and found to have a promoter activity; the sequence displays an array of consensus transcription factor binding sites including MyoD (E box), NF-kB, Spl (GC box) and TATA and CAAT boxes (Hinkes et al, 1993). These observations suggest an association of the signals of bFGF and TGF-β with the promoter activity of syndecan-1 gene. Therefore, PR-39 may also be involved directly or indirectly in the interaction with transcription factors which bind to the promoter sites of syndecan-1 gene or with the signal transduction pathways which are triggered by bFGF and TGF-β.

In conclusion, PR-39 could be a candidate gene to establish a new strategy to inhibit the metastasis of tumour cells because of the unique functions including the induction of syndecan-1, suppression of invasive and motile activities, and change of actin structure.

### ACKNOWLEDGEMENTS

We wish to thank Dr M Bernfield and Dr RL Gallo for providing valuable reagents and for their helpful suggestions. This research was supported by the grants provided by Ministry of Education, Science and Culture, Japan, the Naito Foundation, the Research Fund for Digestive Molecular Biology and the Japanese Fund for Multidisciplinary Treatment of Cancer.

### REFERENCES

Agerberth B, Lee J-Y, Bergman T, Carlquist M, Boman HG, Mutt V and Jornvall H (1991) Amino acid sequence of PR-39: isolation from pig intestine of a new member of the family of proline-arginine-rich antibacterial peptides. *Eur J Biochem* **202**: 849–854

Agerberth B, Gunne H, Odeberg J, Kogner P, Boman HG and Gudmundsson GH (1995) FALL-39, putative human peptide antibiotics, is cysteine-free and expressed in bone marrow and testis. *Proc Natl Acad Sci USA* **92**: 195–199

Agerberth B, Gunne H, Odeberg J, Kogner P, Boman HG and Gudmundsson GH (1996) PR-39, a proline-rich peptide antibiotic from pig, and FALL-39, a tentative human counterpart. *Vet Immunol Immunopathol* **54**: 127–131

Albini A, Iwamoto Y, Kleinmann HK, Martin GR, Aaronson SA, Kozolowski JM and Ecewan RN (1987) A rapid in vitro assay for quantitating the invasive potential of tumor cells. *Cancer Res* **47**: 3239–3245

Albrecht-Buehler G (1977) The phagokinetic tracks of 3T3 cells. *Cell* **11**: 359–404

Aycock RS and Seyer JM (1989) Collagens of normal and cirrhotic human liver. *Connect Tissue Res* **23**: 19–31

Bernfield M, Hinkes MT and Gallo RL (1993) Developmental expression of the syndecans: possible function and regulation. *Development* Suppl: 205–212

 © 1999 Cancer Research Campaign

Boman HG, Agerberth B and Boman A (1993) Mechanism of action on *Escherichia coli* of Cecropin P1 and PR-39, two antibacterial peptides from pig intestine. *Infect Immun* **61**: 2978–2984

Boman HG (1995) Peptide antibiotics and their role in innate immunity. *Annu Rev Immunol* **13**: 61–92

Chan YR and Gallo RL (1998) PR-39, a syndecan-inducing antimicrobial peptide, binds and affects $p130^{Cas}$. *J Biol Chem* **273**: 28978–28985

Dickson RB, Johnson MD, Maemura M and Low J (1996) Anti-invasion drugs. *Breast Cancer Res Treat* **38**: 121–132

Doi I, Namba M and Sato L (1975) Establishment and some biological characteristics of human hepatoma cell lines. *Jpn J Cancer Res* **66**: 385–392

Effert PJ, Gastl G and Strohmeyer T (1996) Current and future strategies to block tumor angiogenesis, invasion, and metastasis. *World J Urol* **14**: 131–140

Elenius K, Salmivirta M, Inki P, Mali M and Jalkanen M (1990) Binding of human syndecan to extracellular matrix proteins. *J Biol Chem* **265**: 17837–17843

Elenius K, Määttä A, Salmivirta M and Jalkanen M (1992) Growth factors induce 3T3 cells to express bFGF-binding syndecan. *J Biol Chem* **267**: 6435–6441

Gallo RL, Ono M, Povsic T, Page C, Eriksson E, Klagsbrunl M and Bernfield M (1994) Syndecan, cell surface heparan sulfate proteoglycans, are induced by a proline-rich antimicrobial peptide from wounds. *Proc Natl Acad Sci USA* **91**: 11035–11039

Gout I, Dhand R, Hiles ID, Fry MJ, Panayotou G, Das P, Truong O, Totty NF, Hsuan J, Booker GW, Campbell ID and Waterfield MD (1993) The GTPase dynamin binds to and is activated by a subset of SH3 domains. *Cell* **75**: 25–36

Hinkes MT, Goldberger OA, Neumann PE, Kokenyesi R and Bernfield M (1993) Organization and promoter activity of the mouse syndecan-1 gene. *J Biol Chem* **268**: 11440–11448

Huh N and Utakoji T (1981) Production of HBs-antigen by two new human hepatoma cell lines and its enhancement by dexamethasone. *Jpn J Cancer Res* **72**: 178–179

Koda JE, Rapraeger A and Bernfield M (1985) Heparan sulfate proteoglycans from mouse mammary epithelial cells. Cell surface proteoglycans as a receptor for interstitial collagens. *J Biol Chem* **260**: 8157–8162

Liebersbach BF and Sanderson RD (1994) Expression of syndecan-1 inhibit cell invasion into type I collagen. *J Biol Chem* **269**: 20013–20019

Martin E, Ganz T and Lehrer RI (1995) Defensins and other endogenous peptide antibiotics of vertebrates. *J Leukocyte Biol* **58**: 128–136

Matsumoto A, Ono M, Fujimoto Y, Gallo RL, Bernfield M and Kohgo Y (1997) Reduced expression of syndecan-1 in human hepatocellular carcinoma with high metastatic potential. *Int J Cancer* **74**: 482–491

Musacchio A, Wilmanns M and Saraste M (1994) Structure and function of the SH3 domain. *Prog Biophys Molec Biol* **61**: 283–297

Pawson T (1995) Protein modules and signaling networks. *Nature* **373**: 573–580

Rapraeger A, Jalkanen M, Endo E, Koda JE and Bernfield M (1985) The cell-surface proteoglycan from mouse mammary epithelial cells bears chondroitin-sulfate and heparan-sulfate GAGs. *J Biol Chem* **260**: 11046–11052

Sanderson RD and Bernfield M (1988) Molecular polymorphism of a cell surface proteoglycan: distinct structures on simple and stratified epithelia. *Proc Natl Acad Sci USA* **85**: 9562–9566

Sanderson RD, Hinkes MT and Bernfield M (1992) Syndecan-1, a cell-surface proteoglycan, changes in size and abundance when keratinocytes stratify. *J Invest Dermatol* **99**: 390–396

Saunders S and Bernfield M (1988) Cell surface proteoglycan binds mouse mammary epithelial cells to fibronectin and behaves as a receptor on interstitial matrix. *J Cell Biol* **106**: 423–430

Saunders S, Jalkanen M, O'Farrell S and Bernfield M (1989) Molecular cloning of syndecan, an integral membrane proteoglycan. *J Cell Biol* **108**: 1547–1556

Shi J, Ross CR, Leto TL and Blecha F (1996) PR-39, a proline-rich antibacterial peptide that inhibits phagocyte NADPH oxidase activity by binding to Src homology 3 domains of $p47^{phox}$. *Proc Natl Acad Sci USA* **93**: 6014–6018

Solursh M, Reiter RS, Jensen KL, Kato M and Bernfield M (1990) Transient expression of a cell surface heparan sulfate proteoglycan (syndecan) during limb development. *Dev Biol* **140**: 83–92

Storici P and Zanetti M (1993) A cDNA derived from pig bone marrow cells predicts a sequence identical to the intestinal antibacterial peptide PR-39. *Biochem Biophys Res Commun* **196**: 1058–1065

Stossel T (1993) On the crawling of animal cells. *Science* **260**: 1086–1094

Vanio S, Lehtonen E, Jalkanen M, Bernfield M and Saxen L (1989) Epithelial–mesenchymal interactions regulate the stage-specific expression of a cell surface proteoglycan, syndecan, in the developing kidney. *Dev Biol* **134**: 382–391

Vainio S, Thesleff I (1992) Coordinated induction of cell proliferation and syndecan expression in dental mesenchyme by epithelium: evidence for diffusible signals. *Dev Dyn* **194**: 105–117

Yeaman C and Rapraeger A (1993) Post-transcriptional regulation of syndecan-1 expression by cAMP in peritoneal macrophages. *J Cell Biol* **122**: 941–950

Yu H, Chen JK, Feng S, Dalgarno DC, Brauer AW and Schreiber SL (1994) Structural basis for the binding of proline-rich peptides to SH3 domains. *Cell* **76**: 933–945

*© 1999 Cancer Research Campaign*
